# LINC00426 is a potential immune phenotype-related biomarker and an overall survival predictor in PAM50 luminal B breast cancer

**DOI:** 10.3389/fgene.2023.1034569

**Published:** 2023-05-16

**Authors:** Marco Antonio Fonseca-Montaño, Mireya Cisneros-Villanueva, Isabelle Coales, Alfredo Hidalgo-Miranda

**Affiliations:** ^1^ Laboratorio de Genómica del Cáncer, Instituto Nacional de Medicina Genómica (INMEGEN), Mexico City, Mexico; ^2^ Programa de Doctorado, Posgrado en Ciencias Biológicas, Unidad de Posgrado, Universidad Nacional Autónoma de México (UNAM), Mexico City, Mexico; ^3^ Centre for Host Microbiome Interactions, King’s College London, London, United Kingdom

**Keywords:** breast cancer, PAM50 subtypes, luminal B, LINC00426, immune-cell infiltration, immune checkpoint genes, cytolytic activity-related genes, tumor immune microenvironment

## Abstract

**Background:** Breast cancer (BRCA) represents the most frequent diagnosed malignancy in women worldwide. Despite treatment advances, BRCAs eventually develop resistance to targeted therapies, resulting in poor prognosis. The identification of new biomarkers, like immune-related long non-coding RNAs (lncRNAs), could contribute to the clinical management of BRCA patients. In this report, we evaluated the LINC00426 expression in PAM50 BRCA subtypes from two clinical independent cohorts (BRCA-TCGA and GEO-GSE96058 datasets).

**Methods and results:** Using Cox regression models and Kaplan-Meier survival analyses, we identified that LINC00426 expression was a consistent overall survival (OS) predictor in luminal B (LB) BRCA patients. Subsequently, differential gene expression and gene set enrichment analyses identified that LINC00426 expression was associated with different immune-related and cancer-related pathways and processes in LB BRCA. Additionally, the LINC00426 expression was correlated with the infiltration level of diverse immune cell populations, alongside immune checkpoint and cytolytic activity-related gene expression.

**Conclusion:** This evidence suggests that LINC00426 is a potential biomarker of immune phenotype and an OS predictor in PAM50 LB BRCA.

## 1 Introduction

In 2020, breast cancer (BRCA) was the most frequent diagnosed malignancy and the leading cause of cancer-related death in women worldwide (Harbeck et al., 2019; Sung et al., 2021). BRCA is a heterogeneous disease which includes well-defined histological types and protein markers, such as estrogen receptor (ER), progesterone receptor (PR), human epidermal growth factor receptor 2 (HER2) and Ki-67 (Bydoun et al., 2013; Goldhirsch et al., 2013; Ignatiadis and Sotiriou, 2013; Akram et al., 2017; Harbeck et al., 2019). According to the PAM50 gene signature, BRCA is classified in four intrinsic molecular subtypes: Luminal A (LA), Luminal B (LB), HER2-enriched and Basal-like (BL) (Perou et al., 2000; Bernard et al., 2009; Gao and Swain, 2018; Harbeck et al., 2019). In contrast to HER2-enriched and BL subtypes, luminal BRCAs constitute around 60%–70% of diagnosed cases and are commonly associated with improved clinical outcomes (Perou et al., 2000; Bernard et al., 2009; Harbeck et al., 2019). Despite treatment advances, BRCAs eventually develop resistance to therapies due to mutations and dysregulations in diverse genes and signaling pathways (Goldhirsch et al., 2013; Ignatiadis and Sotiriou, 2013; Akram et al., 2017; Brufsky and Dickler, 2018; Rani et al., 2019; Li et al., 2020a; Han et al., 2020; Hartkopf et al., 2020; Luque-Bolivar et al., 2020; Marra et al., 2020; Prat et al., 2020). The identification of new prognostic biomarkers and therapeutic targets, like long non-coding RNAs (lncRNAs), is a new area of research that could contribute to the clinical management of BRCA patients (Xu et al., 2017; Wang L. et al., 2020; Zhang L. et al., 2020; Cedro-Tanda et al., 2020; Ríos-Romero et al., 2020; Cisneros-Villanueva et al., 2021).

LncRNAs are a class of non-protein-coding transcripts greater than 200 nucleotides in length. Within a cell, lncRNAs are key players in a wide range of biological functions like regulation of gene expression, chromatin modification, genomic imprinting, transcriptional and translational processing (Zhu et al., 2013; Chen, 2016). Previous investigations showed that dysregulation in lncRNAs is associated with progression in diverse cancer types (Qiu et al., 2013; Bhan et al., 2017; Bolha et al., 2017; Schmitt and Chang, 2017), including recent studies that have demonstrated the association of different lncRNAs in processes related with cancer immunobiology, such as antigen presentation, immune cell infiltration and functional modulation of immune cells in the tumor immune microenvironment (TIME) (Denaro et al., 2019; Zhang Y. et al., 2020; Luo et al., 2020; Wu et al., 2020). The relevance of some immune-related lncRNAs in BRCA has been explored (Lin et al., 2016; Huang et al., 2018; Pei et al., 2018; Zhang L. et al., 2020; Liu et al., 2020). However, the role of diverse lncRNAs in BRCA immunobiology is unknown.

*LINC00426* is a human lncRNA gene which contains 38,105 bases in length and is in the 13q12.3 region of the DNA antisense strand (GeneCards, 2021). LINC00426 is known to be associated with lung adenocarcinoma (LUAD) progression (Li H. et al., 2020), doxorubicin resistance in osteosarcoma (OSA) (Wang L. et al., 2020), immune-cell infiltration in clear cell renal cell carcinoma (ccRCC) (Xiang et al., 2021) and prognosis in hepatocellular carcinoma (HCC) patients (Zhu et al., 2020). Moreover, high expression of LINC00426 is associated with improved overall survival (OS) in non-small cell lung cancer (NSCLC) and LUAD (Du, 2020). In contrast, the high expression of this lncRNA is related to poor OS in OSA patients (Wang Y. et al., 2020). Despite these findings, the prognostic and biological role of LINC00426 in PAM50 BRCA subtypes remains unknown.

We evaluated the LINC00426 expression in PAM50 BRCA subtypes through RNA-seq data from two clinical independent cohorts (BRCA-TCGA and GEO-GSE96058 datasets) of public databases. Using Cox regression models and Kaplan-Meier survival analyses, we found that LINC00426 expression is associated with OS in LB BRCA patients from both cohorts. Differential gene expression (DGE) and gene set enrichment analyses (GSEA) revealed that LINC00426 is associated with different immune-related and cancer-related pathways and processes in LB BRCA. Additionally, the LINC00426 expression correlates with immune-cell infiltration, expression of immune checkpoint genes (ICG) and cytolytic activity-related genes (CARG). These data suggest that LINC00426 is a potential biomarker of immune phenotype and an OS predictor in PAM50 LB BRCA.

## 2 Materials and methods

### 2.1 Collection of BRCA clinical datasets (TCGA and GEO databases) and stratification according with the LINC00426 expression

Clinical information and raw RNA-seq expression data from BRCA patients in different PAM50 subtypes were obtained from TCGA database through cBioPortal (https://www.cbioportal.org/) and GDC Data Portal (https://portal.gdc.cancer.gov/projects). Female patients who received neoadjuvant treatment and/or were lacking OS data were excluded, leaving a total of 927 patients analyzed in this study (LA: *n* = 490; LB: *n* = 192; HER2-enriched: *n* = 77; and BL: *n* = 168). The raw expression data were normalized to transcripts per million (TPM) and log_2_(TPM+1). A validation cohort (GSE96058) of 3,052 patients (LA: *n* = 1,657; LB: *n* = 729; HER2-enriched: *n* = 327; and BL: *n* = 339) was obtained from GEO database (https://www.ncbi.nlm.nih.gov/geo/query/acc.cgi?acc=GSE96058). Again, female patients were lacking OS data and/or samples with label “repl” were excluded. For both datasets, patients were stratified in groups of low and high expression of LINC00426 by PAM50 BRCA subtypes, based on the lower (25%) and upper quartile (75%), respectively.

### 2.2 Survival analyses, number at risk by time and Cox proportional hazards regression analyses

Considering the OS data (in months), Kaplan-Meier survival analyses were performed through the log-rank test in patients stratified by PAM50 BRCA subtypes, according to the low and high expression of LINC00426. These analyses were performed using the R packages *survival* (version 3.4.0) and *survminer* (version 0.4.9). The absolute number of patients at risk by time (in months) was determined through the *survfit()* command and *n.risk* option from *survival* (version 3.4.0). Univariate analyses were performed through Cox proportional hazards regression models to identify clinicopathological variables associated with OS of patients stratified by PAM50 BRCA subtypes. Multivariate analyses were performed using Cox proportional hazards regression models and OS predictor variables, statistically significant, obtained via univariate analyses. Hazard ratios (HR) and 95% confidence intervals were obtained for each clinicopathological variable. These analyses were performed via *survival* (version 3.4.0) and *survminer* (version 0.4.9). *p* values < 0.05 were considered statistically significant.

### 2.3 Differential gene expression and functional annotation

DGE between groups of BRCA patients with low and high expression of LINC00426 was determined using the R package *DESeq2* (version 1.38.1) (Love et al., 2014). Raw counts less than 10 were filtered and differentially expressed genes were defined as those with log_2_FoldChange (LFC) > 1.5 and <−1.5 with adjusted *p* values < 0.05. Volcano plots were generated using the R package *EnhancedVolcano* (version 1.16.0). The list of differentially expressed genes was used to perform Gene Ontology (GO) over-representation analyses for biological processes and molecular functions, using the R package *clusterProfiler* (version 4.6.0) (Yu et al., 2012). Kyoto Encyclopedia of Genes and Genomes (KEGG) pathway analysis was performed to identify signaling pathways related with the LINC00426 expression through *clusterProfiler* using the default parameters. The results were represented as dot plots and adjusted *p* values < 0.05 were considered statistically significant.

### 2.4 Gene set enrichment analysis

Using the normalized RNA-seq expression data from groups of patients with low and high expression of LINC00426, a GSEA (Subramanian et al., 2005) was performed using the Hallmarks gene sets in *GSEA* (version 4.1.0) with default parameters and 1,000 permutations. Gene sets with nominal *p* values < 0.05 and FDR <25% were considered statistically significant, according to the GSEA User Guide instructions for sample size and phenotype permutations (https://www.gsea-msigdb.org/gsea/doc/GSEAUserGuideFrame.html?Interpreting_GSEA).

### 2.5 Estimation of tumor-infiltrating immune cell populations

Using CIBERSORTx, an analytical software based on transcriptome deconvolution method to infer the cell-type-specific gene expression and cell type abundance from RNA-seq data, the relative abundance of 22 tumor-infiltrating immune cell populations was determined: naive B cells, memory B cells, plasma cells, CD8 T cells, naive CD4 T cells, memory CD4 T cells (resting), memory CD4 T cells (activated), T follicular helper cells, regulatory T cells, gamma-delta T cells, NK cells (resting), NK cells (activated), monocytes, M0 macrophages, M1 macrophages, M2 macrophages, dendritic cells (resting), dendritic cells (activated), mast cells (resting), mast cells (activated), eosinophils and neutrophils (Chen B. et al., 2018; Newman et al., 2019; https://cibersortx.stanford.edu/). This analysis was performed using the normalized gene expression data with 1,000 permutations. *p* values < 0.05 were considered statistically significant.

### 2.6 ICG and CARG expression signatures

The ICG expression signature for each patient was determined by calculating the geometric mean of seven ICGs (*PDCD1, PDCD1LG2, CD274, CTLA4, LAG3, TIGIT* and *IDO1*) (Haddad et al., 2020). Similarly, the CARG expression signature for each patient was determined by the geometric mean of three CARGs (*GZMA, GZMB* and *PRF1*) (Rooney et al., 2015).

### 2.7 Pan-cancer OS analysis in GEPIA2 platform

We used GEPIA2 to analyze OS based on LINC00426 expression across 32 cancer types (http://gepia2.cancer-pku.cn/#general) (Tang et al., 2019). The OS heatmap with HRs, was generated considering the lower (25%) and upper quartile (75%) for each cancer type. The OS contribution related with the LINC00426 expression was estimated through the Mantel-Cox test and adjusted *p* values < 0.05 were considered statistically significant. The 32 cancer types included in this analysis are as follows: acute myeloid leukemia, adrenocortical carcinoma, bladder urothelial carcinoma, cervical squamous cell carcinoma and endocervical adenocarcinoma, cholangiocarcinoma, colon adenocarcinoma, diffuse large B-cell lymphoma, esophageal carcinoma, glioblastoma multiforme, head and neck squamous cell carcinoma, kidney chromophobe, kidney renal clear cell carcinoma, kidney renal papillary cell carcinoma, low grade glioma, liver hepatocellular carcinoma, lung adenocarcinoma, lung squamous cell carcinoma, mesothelioma, ovarian serous cystadenocarcinoma, pancreatic adenocarcinoma, pheochromocytoma and paraganglioma, prostate adenocarcinoma, rectum adenocarcinoma, sarcoma, skin cutaneous melanoma, stomach adenocarcinoma, testicular germ cell tumors, thyroid carcinoma, thymoma, uterine corpus endometrial carcinoma, uterine carcinosarcoma and uveal melanoma.

### 2.8 Statistical analyses

Statistical analyses were performed through *GraphPad Prism* (version 8.3.0) and R package *ggpubr* (version 0.5.0). The non-parametric Kruskal-Wallis test was performed to identify differences in the LINC00426 expression between PAM50 BRCA subtypes. The non-parametric Mann-Whitney U test was performed to determine differences in the infiltration level of immune cell populations, ICG and CARG expression between groups of BRCA patients with low and high expression of LINC00426. Spearman correlation analyses were performed to determine statistical relationships between the LINC00426 expression and the infiltration level of immune cell populations, ICG and CARG expression (as individual genes and as gene expression signatures). *p* values < 0.05 were considered statistically significant.

## 3 Results

### 3.1 The LINC00426 expression is an OS prognostic marker in PAM50 LB BRCA patients

**FIGURE 1:**
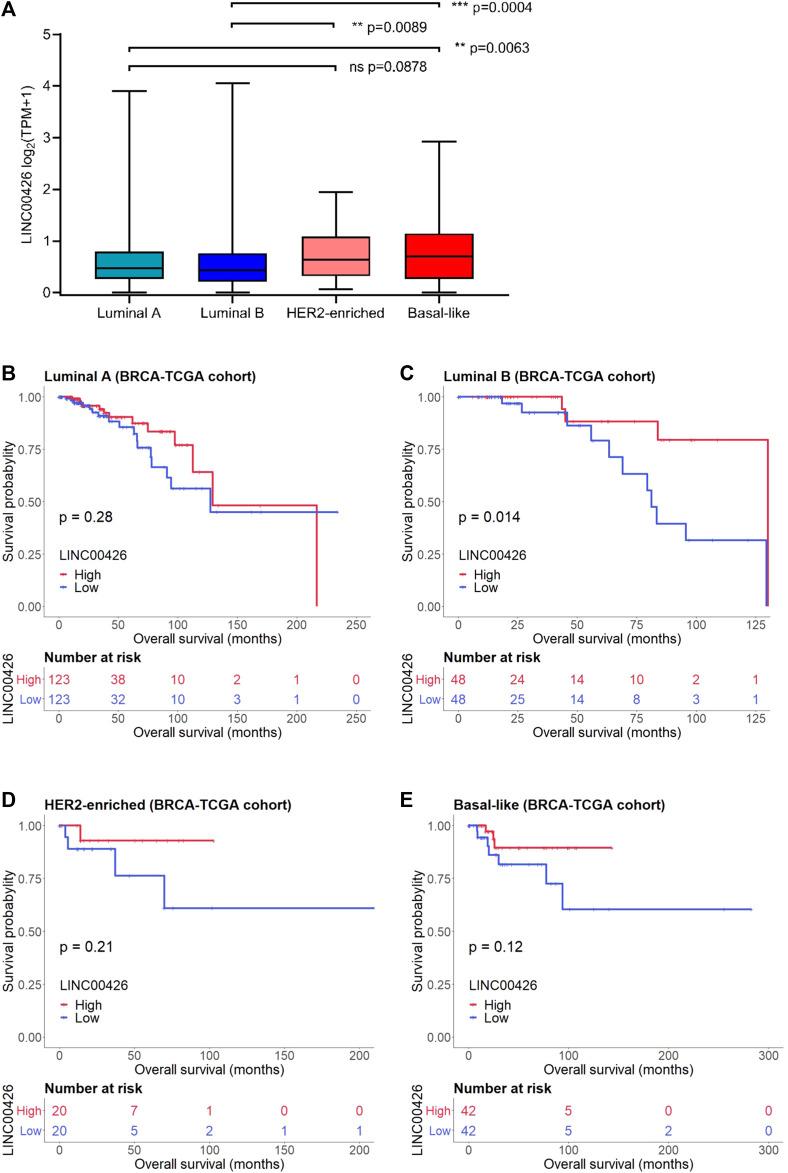
LINC00426 expression and Kaplan-Meier survival analyses in PAM50 patients from the BRCA-TCGA cohort. **(A)** Expression level of LINC00426 in BRCA patients stratified by PAM50 subtypes (Kruskal-Wallis test) (luminal A: *n* = 490; luminal B: *n* = 192; HER2-enriched: *n* = 77; and basal-like: n = 168). **(B)** Kaplan-Meier survival plot of LINC00426 expression in the OS of luminal A (*n* = 490), **(C)** luminal B (*n* = 192), **(D)** HER2-enriched (*n* = 77), **(E)** basal-like (*n* = 168) BRCA patients. The high and low expression of LINC00426 show associations with increased and reduced OS in PAM50 luminal B BRCA patients (*p* < 0.05), respectively.

Analysis of the BRCA-TCGA cohort (*n* = 927) found high expression of LINC00426 in non-luminal compared to luminal BRCA subtypes. Significant differences were identified between LA and BL (*p* = 0.0063), LB and HER2-enriched (*p* = 0.0089), LB and BL (*p* = 0.0004) subtypes. No significant difference was observed in the LINC00426 expression between LA and HER2-enriched subtypes (*p* = 0.0878) (Figure 1A). The clinicopathological characteristics of BRCA-TCGA patients are described in Table 1. Next, we evaluated whether the LINC00426 expression is an OS prognostic marker in patients across different BRCA subtypes. Kaplan-Meier survival analyses revealed that LINC00426 expression did not have an OS prognostic value in LA (*p* = 0.28), HER2-enriched (*p* = 0.21) and BL BRCA patients (*p* = 0.12) (Figures 1B, D, E). However, the high and low expression of LINC00426 were associated with increased and reduced OS in LB BRCA patients (*p* = 0.01) (Figure 1C), respectively. Univariate Cox regression analyses showed that LINC00426 expression was an OS predictor in LB BRCA patients (*p* = 0.024), with no significance observed in LA (*p* = 0.288), HER2-enriched (*p* = 0.246) and BL (*p* = 0.133) subtypes (Table 2). The OS prognostic value of LINC00426 expression in LB subtype remained significant on multivariate Cox regression analysis (Table 3). These results suggest that LINC00426 expression has a subtype-specific and an OS prognostic value in PAM50 LB BRCA patients from the TCGA cohort. Also, we identified that age (≤58 years old) was a good prognostic factor associated with OS and that late tumor stage, positive lymph node status and positive metastasis status were poor prognostic factors associated with OS in LA BRCA patients. These clinicopathological variables were also prognostic factors associated with OS in LB, HER2-enriched and BL BRCA patients (Table 2).

**TABLE 1:** Clinicopathological characteristics of luminal A, luminal B, HER2-enriched and basal-like patients from the BRCA-TCGA cohort (*n* = 927).

		Luminal A (*n* = 490)	Luminal B (*n* = 192)	HER2-enriched (*n* = 77)	Basal-like (*n* = 168)
Variable	Stratification	Frequency (n)	Frequency (n)	Frequency (n)	Frequency (n)
Age	≤58	221	99	44	103
	>58	269	93	33	65
Tumor stage	I-II	366	129	53	141
	III-IV	110	61	22	23
	NA	14	2	2	4
Lymph node status	Positive	249	109	46	64
	Negative	231	79	27	104
	NA	10	4	4	0
Metastasis status	Positive	8	4	3	3
	Negative	402	166	66	147
	NA	80	22	8	18
ER status	Positive	460	177	26	20
	Negative	11	3	45	141
	NA	19	12	6	7
PR status	Positive	424	145	13	11
	Negative	44	35	60	148
	NA	22	12	4	9
HER2 status	Positive	54	30	47	9
	Negative	268	91	13	102
	NA	168	71	17	57
OS status	Alive	434	161	62	146
	Dead	56	31	15	22

BRCA, breast cancer; ER, estrogen receptor; HER2, human epidermal growth factor receptor 2; NA, not available; OS, overall survival; PR, progesterone receptor.

**TABLE 2:** Univariate Cox proportional hazard regression analyses of clinicopathological variables impacting in the OS of luminal A, luminal B, HER2-enriched and basal-like BRCA patients (BRCA-TCGA cohort), including the LINC00426 expression.

Variables	Luminal A (n = 490)		Luminal B (n = 192)	
	HR (95% CI)	*p*-value	HR (95% CI)	*p*-value
Age (≤58)	**0.519 (0.301–0.895)**	**0.018**	0.612 (0.297–1.262)	0.183
Tumor stage (late)	**2.072 (1.161–3.697)**	**0.014**	**2.101 (1.011–4.365)**	**0.047**
Lymph node status (positive)	**1.844 (1.038–3.278)**	**0.037**	2.079 (0.949–4.554)	0.067
Metastasis status (positive)	**6.045 (2.527–14.460)**	**< 0.001**	3.048 (0.881–10.550)	0.078
ER status (positive)	0.745 (0.230–2.408)	0.622	1.852 (0.238–14.390)	0.556
PR status (positive)	0.829 (0.374–1.834)	0.643	1.055 (0.424–2.623)	0.907
HER2 status (positive)	2.025 (0.815–5.029)	0.129	1.636 (0.455–5.879)	0.451
LINC00426 expression (low)	1.480 (0.718–3.050)	0.288	**4.387 (1.217–15.810)**	**0.024**

Variables	HER2-enriched (n = 77)		Basal-like (n = 168)	
	HR (95% CI)	*p*-value	HR (95% CI)	*p*-value
Age (≤58)	**0.163 (0.044–0.603)**	**0.006**	0.888 (0.371–2.125)	0.790
Tumor stage (late)	**4.859 (1.572–15.020)**	**0.006**	**6.045 (2.457–14.870)**	**< 0.001**
Lymph node status (positive)	3.056 (0.659–14.180)	0.154	**4.385 (1.714–11.220)**	**0.002**
Metastasis status (positive)	**13.620 (3.485–53.270)**	**< 0.001**	3.671 (0.708–19.020)	0.121
ER status (positive)	1.368 (0.417–4.490)	0.606	0.251 (0.033–1.888)	0.179
PR status (positive)	0.653 (0.143–2.986)	0.582	1.344 × 10^−8^ (0.0-Inf)	0.998
HER2 status (positive)	0.513 (0.130–2.021)	0.340	3.206 (0.680–15.110)	0.141
LINC00426 expression (low)	3.665 (0.409–32.860)	0.246	2.822 (0.729–10.930)	0.133

Bold indicates *p* values < 0.05. A variable with HR < 1 indicates a poor prognostic factor, while a variable with HR > 1 indicates a good prognostic factor.

BRCA, breast cancer; CI, confidence interval; HR, hazard ratio.

**TABLE 3:** Multivariate Cox proportional hazard regression analysis of clinicopathological variables impacting in the OS of luminal B BRCA patients (BRCA-TCGA cohort).

Variables	Luminal B (*n* = 192)	
	HR (95% CI)	*p*-value
Tumor stage (late)	2.643 (0.837–8.351)	0.098
**LINC00426 expression (low)**	**4.587 (1.229–17.119)**	**0.023**

Bold indicates *p* values < 0.05. A variable with HR < 1 indicates a poor prognostic factor, while a variable with HR > 1 indicates a good prognostic factor.

BRCA, breast cancer; CI, confidence interval; HR, hazard ratio.

### 3.2 LINC00426 is associated with different immune-related and cancer-related processes

**FIGURE 2:**
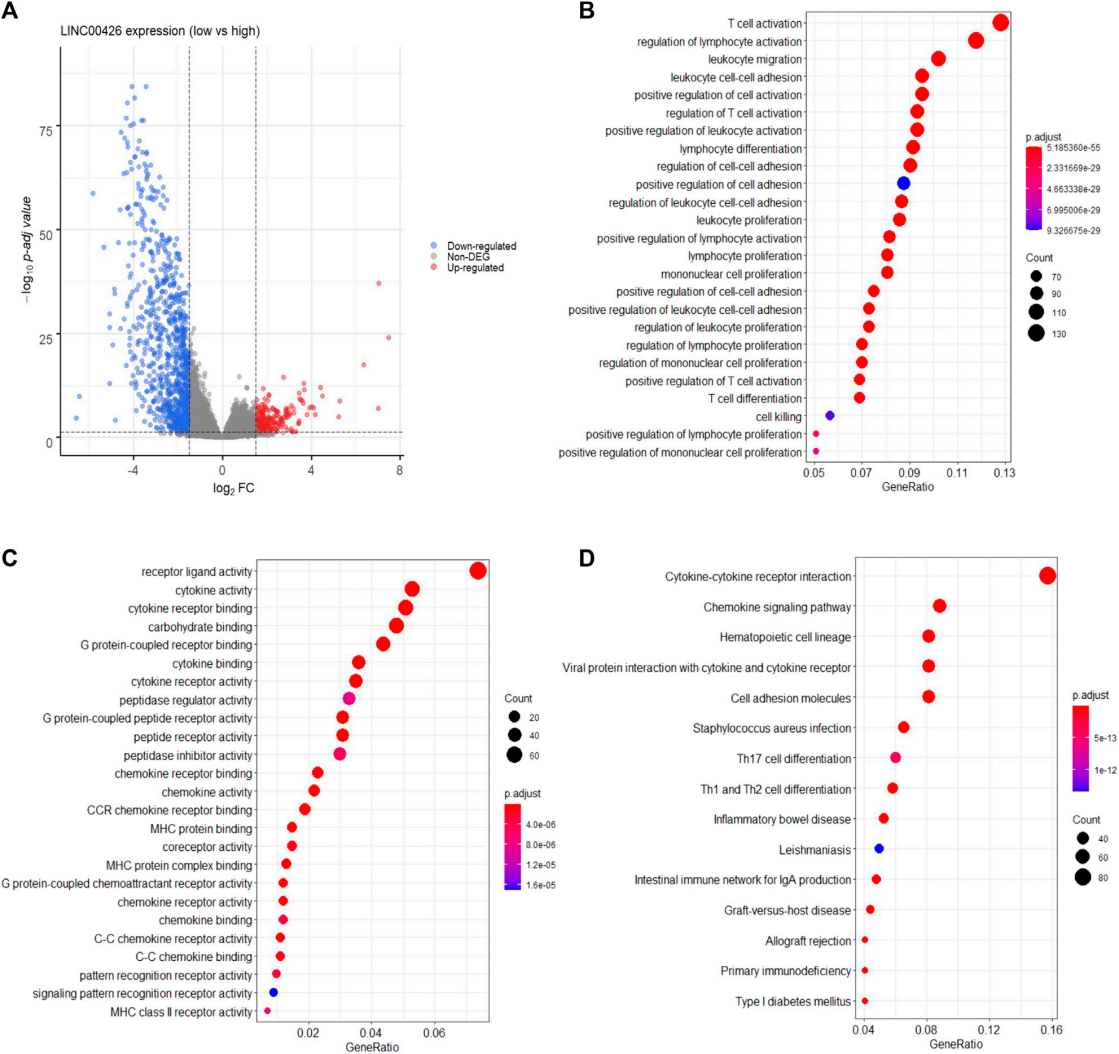
Functional annotation of LINC00426 in PAM50 LB patients from the BRCA-TCGA cohort. **(A)** Volcano plot shows 1,139 differentially expressed genes between PAM50 LB BRCA patients with low and high expression of LINC00426. **(B)** GO analysis dot plot shows the over-representation of immune-related biological processes, and **(C)** immune-related molecular functions. **(D)** KEGG analysis shows the enrichment of immune-related signaling pathways.

Next, we aimed to identify the biological processes and molecular functions associated with the LINC00426 expression in PAM50 LB BRCA through DGE analysis of protein-coding genes between patients with low and high expression of LINC00426. A total of 1,139 genes were found to be differentially expressed, of which 915 genes were downregulated (i.e., *CLEC6A, IFNG, PLA2G2D, DCD* and *GNAT3*) and 224 genes were upregulated (i.e., *CPB1, TRH, SYT4, CPLX2* and *NELL1*) (Figure 2A; Supplementary Table S1). Subsequent GO analysis of the differentially expressed genes identified a significant over-representation of diverse immune-related biological processes, including the activation, migration, differentiation, proliferation, and cell-cell adhesion of T cells, lymphocytes and leukocytes (Figure 2B), alongside the over-representation of immune-related molecular functions, including signaling via cytokines, chemokines and MHC proteins (adjusted *p*-value < 0.05) (Figure 2C). The KEGG pathway analysis corroborated that LINC00426 is associated with immune-related signaling pathways (i.e., cytokine-cytokine receptor interaction, chemokine signaling pathways, hematopoietic cell linage and cell adhesion molecules) (adjusted *p*-value < 0.05) (Figure 2D).

The GSEA, showed that the group of LB BRCA patients with high expression of LINC00426 is significatively enriched (nominal *p*-value < 0.05 and FDR <25%) with eight immune-related gene sets (IL2-STAT5 signaling, complement, inflammatory response, allograft rejection, IL6-JAK-STAT signaling, interferon gamma response, TNFα signaling via NFκB and interferon alpha response) and seven cancer-related gene sets (KRAS signaling up, apoptosis, coagulation, epithelial-mesenchymal transition, PI3K-AKT-mTOR, apical junction and apical surface) (Table 4). In contrast, two cancer-related gene sets (estrogen response late and DNA repair) were significatively enriched in the group of LB BRCA patients with low expression of LINC00426 (nominal *p*-value < 0.05 and FDR <25%) (Table 5). Altogether, these results suggest that LINC00426 could play an important role in the regulation of LB BRCA immunobiology.

**TABLE 4:** GSEA and statistical values for enriched hallmark gene sets in PAM50 LB BRCA patients with high expression of LINC00426 (BRCA-TCGA cohort).

Hallmark gene set	Function	ES	NES	Nominal *p*-value	FDR value
IL2_STAT5_SIGNALING	Immune-related	0.616	2.558	0.000	0.000
COMPLEMENT	Immune-related	0.633	2.554	0.000	0.000
INFLAMMATORY_RESPONSE	Immune-related	0.718	2.506	0.000	0.000
ALLOGRAFT_REJECTION	Immune-related	0.808	2.459	0.000	0.000
KRAS_SIGNALING_UP	Cancer-related	0.606	2.393	0.000	0.000
IL6_JAK_STAT3_SIGNALING	Immune-related	0.725	2.333	0.000	0.000
INTERFERON_GAMMA_RESPONSE	Immune-related	0.767	2.272	0.002	0.000
TNFA_SIGNALING_VIA_NFKB	Immune-related	0.610	2.258	0.000	0.000
APOPTOSIS	Cancer-related	0.451	2.047	0.000	0.003
INTERFERON_ALPHA_RESPONSE	Immune-related	0.743	2.040	0.002	0.003
COAGULATION	Cancer-related	0.532	2.039	0.002	0.003
EPITHELIAL_MESENCHYMAL_TRANSITION	Cancer-related	0.622	1.942	0.015	0.008
PI3K_AKT_MTOR_SIGNALING	Cancer-related	0.387	1.753	0.004	0.028
APICAL_JUNCTION	Cancer-related	0.396	1.740	0.006	0.030
APICAL_SURFACE	Cancer-related	0.392	1.470	0.043	0.138

Gene sets with nominal *p* values < 0.05 and FDR <25% are considered statistically significant.

ES, enrichment score; NES, normalized enrichment score.

**TABLE 5:** GSEA and statistical values for enriched hallmark gene sets in PAM50 LB BRCA patients with low expression of LINC00426 (BRCA-TCGA cohort).

Hallmark gene set	Function	ES	NES	Nominal *p*-value	FDR value
ESTROGEN_RESPONSE_LATE	Cancer-related	−0.364	−1.604	0.004	0.231
DNA_REPAIR	Cancer-related	−0.413	−1.578	0.040	0.203

Gene sets with nominal *p* values < 0.05 and FDR <25% are considered statistically significant.

ES, enrichment score; NES, normalized enrichment score.

### 3.3 The LINC00426 expression shows differences in the infiltration level of immune cell populations

**FIGURE 3:**
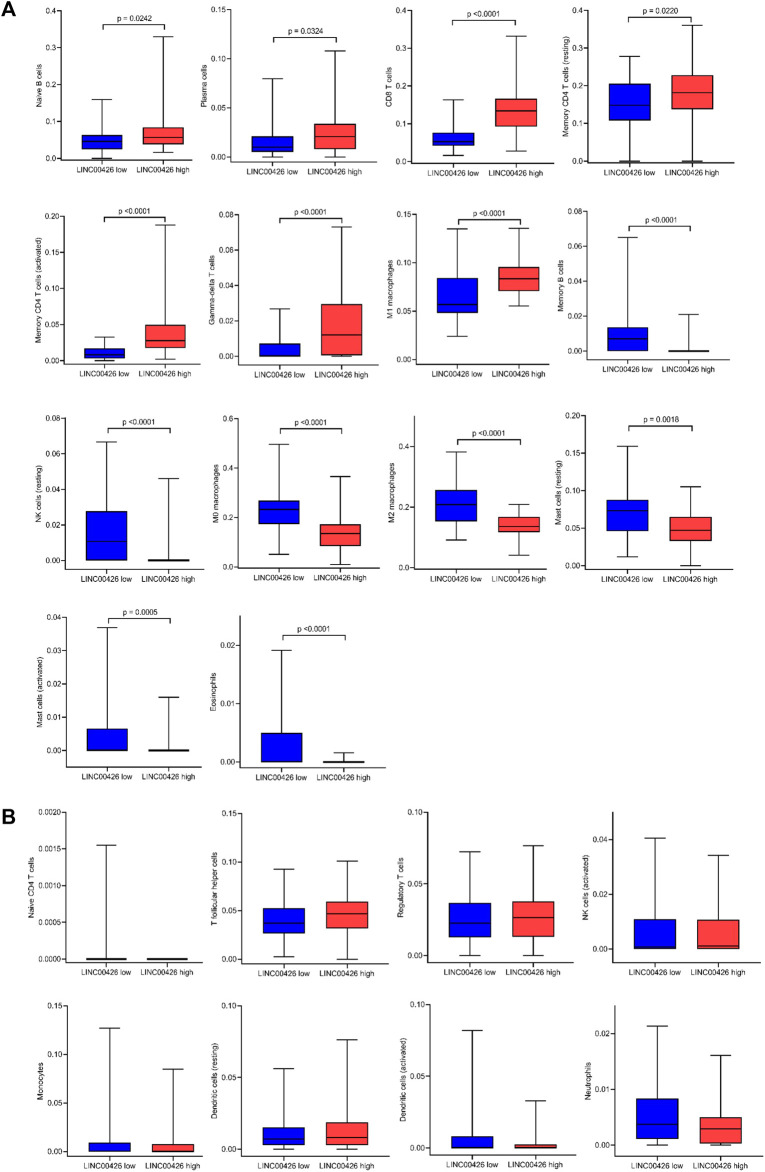
Infiltration level of immune cell populations in PAM50 LB patients with low and high expression of LINC00426 from the BRCA-TCGA cohort. **(A)** Mann-Whitney U test shows significant differences (*p* < 0.05) in the infiltration level of naive B cells, plasma B cells, CD8 T cells, memory CD4 T cells (resting), memory CD4 T cells (activated), gamma-delta T cells, M1 macrophages, memory B cells, NK cells (resting), M0 macrophages, M2 macrophages, mast cells (resting), mast cells (activated) and eosinophils. **(B)** Infiltration of naive CD4 T cells, T follicular helper cells, regulatory T cells, NK cells (activated), monocytes, dendritic cells (resting), dendritic cells (activated) and neutrophils did not show significant differences (Mann-Whitney U test, *p* > 0.05).

After identifying that LINC00426 is associated with immune-related and cancer-related processes, we used CIBERSORTx to estimate the abundance of 22 tumor-infiltrating immune cell populations (Chen C. et al., 2018; Newman et al., 2019) in groups of patients with low and high expression of LINC00426 in the PAM50 LB subtype from the BRCA-TCGA cohort. Using Spearman correlation analyses, we found that LINC00426 expression differentially correlates with the infiltration of 15 immune cell populations (Supplementary Table S2). LB BRCA patients with low expression of LINC00426 have reduced infiltration of naive B cells (*p* = 0.0242), plasma cells (*p* = 0.0324), CD8 T cells (*p* < 0.0001), memory CD4 T cells (resting) (*p* = 0.0220), memory CD4 T cells (activated) (*p* < 0.0001), gamma-delta T cells (*p* < 0.0001), M1 macrophages (*p* < 0.0001) and increased infiltration of memory B cells (*p* < 0.0001), NK cells (resting) (*p* < 0.0001), M0 macrophages (*p* < 0.0001), M2 macrophages (*p* < 0.0001), mast cells (resting) (*p* = 0.0018), mast cells (activated) (*p* = 0.0005) and eosinophils (*p* < 0.0001). These immune cell infiltration patterns are reverted in patients with high expression of LINC00426 (Figure 3A). The infiltration of naive CD4 T cells, T follicular helper cells, regulatory T cells, NK cells (activated), monocytes, dendritic cells (resting), dendritic cells (activated) and neutrophils did not show significant differences between groups of patients with low and high expression of LINC00426 (*p* > 0.05) (Figure 3B). These results suggest that PAM50 LB BRCA patients with low expression of LINC00426 are enriched with immune cell populations associated with immune evasion, which could be related with immunosuppressive TIMEs. Conversely, PAM50 LB BRCA patients with high expression of LINC00426 are enriched with anti-tumoral immune cell populations, which could be associated with inflammatory TIMEs.

### 3.4 The LINC00426 expression positively correlates with ICG and CARG expression

**FIGURE 4:**
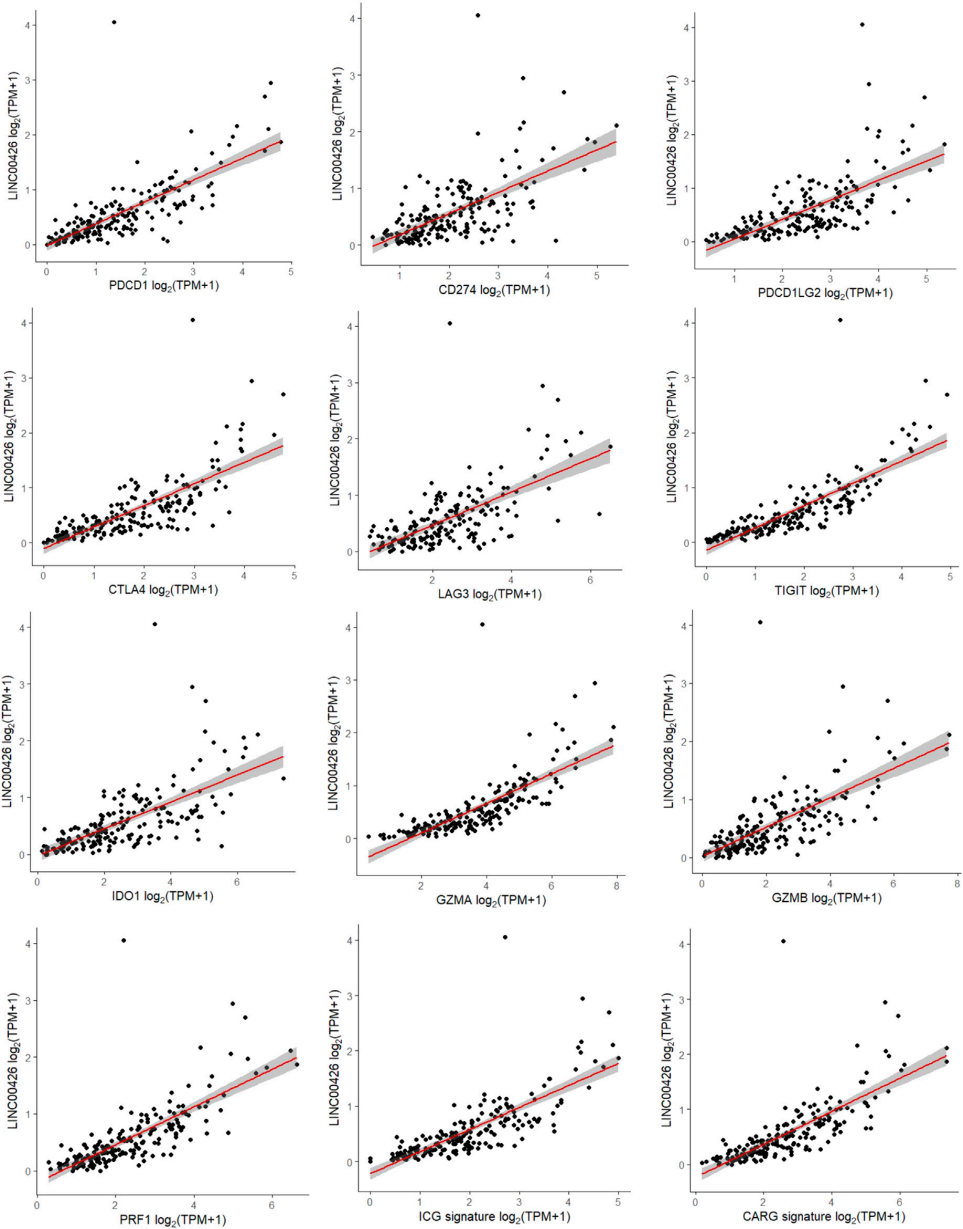
Spearman correlation analyses of the LINC00426 expression with ICG and CARG expression in PAM50 LB patients from the BRCA-TCGA cohort. Positive correlation with statistical significance (*p* < 0.001) was identified in *PDCD1, CD274, PDCD1LG2, CTLA4, LAG3, TIGIT, IDO1, GZMA, GZMB, PRF1,* ICG and CARG signatures.

**FIGURE 5:**
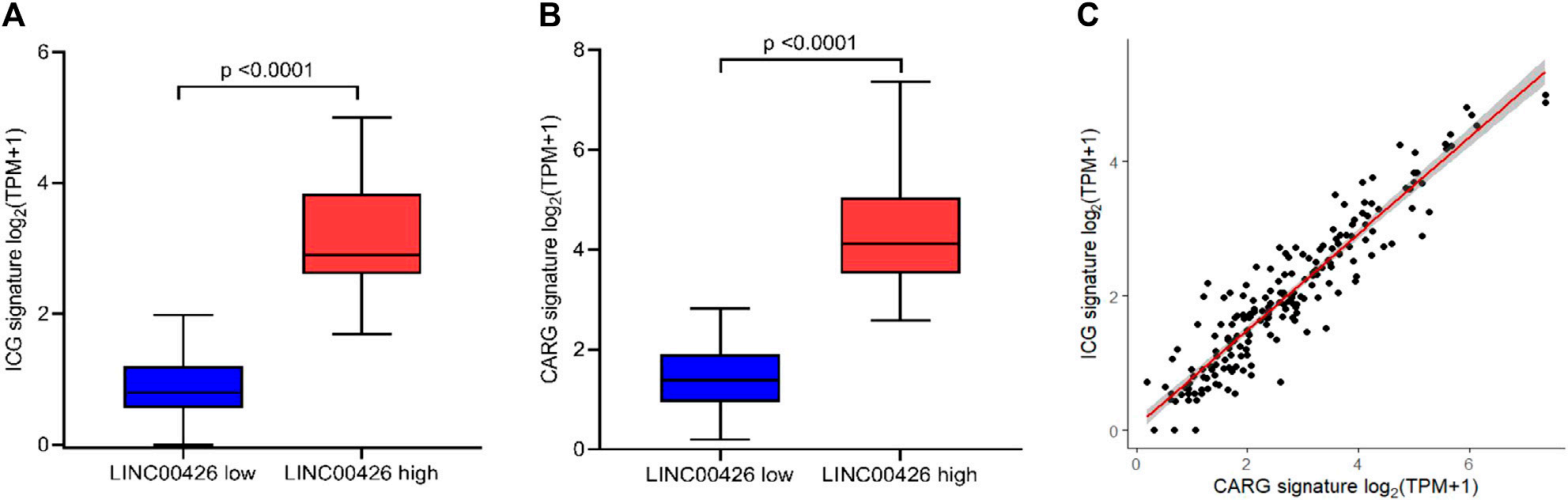
ICG and CARG signature expression in PAM50 LB patients from the BRCA-TCGA cohort. Differences in the **(A)** ICG and **(B)** CARG signature expression were detected between groups of patients with low and high expression of LINC00426 (*p* < 0.0001). **(C)** Spearman correlation between ICG and CARG signature expression in PAM50 LB BRCA patients (*p* < 0.001).

The evaluation of markers associated with the functional status of immune cells in the TIME is useful to determine the tumor immune status and for immunotherapies selection in patients. Well-defined markers associated to immune checkpoint and cytolytic activity are frequently used in immuno-oncology (Rooney et al., 2015; Sharma and Allison, 2015; Narayanan et al., 2018; He and Xu, 2020). Since previous studies suggest that some lncRNAs could be related with these immune-functional markers in cancer (Peng et al., 2020; Salama et al., 2020; Samir et al., 2021), we evaluated whether the LINC00426 expression correlates with ICGs (*PDCD1, PDCD1LG2, CD274, CTLA4, LAG3, TIGIT* and *IDO1*) and CARGs (*GZMA, GZMB* and *PRF1*) expression in the PAM50 LB subtype from the BRCA-TCGA cohort. Spearman correlation analyses indicated that LINC00426 expression, positively and significatively, correlates with *PDCD1* (R = 0.797), *PDCD1LG2* (R = 0.736), *CD274* (R = 0.618), *CTLA4* (R = 0.821), *LAG3* (R = 0.639), *TIGIT* (R = 0.901), *IDO1* (R = 0.738), *GZMA* (R = 0.878), *GZMB* (R = 0.749), *PRF1* (R = 0.830), ICG (R = 0.843) and CARG signatures (R = 0.845) (*p* < 0.001) (Figure 4). Concordantly, the expression of ICG and CARG signatures were found to be significantly decreased in LB BRCA patients with low expression of LINC00426, in contrast to the high expression group (*p* < 0.0001) (Figures 5A, B). Further analysis indicated that the expression of ICG and CARG signatures were positively correlated in LB BRCA (*p* < 0.001) (Figure 5C). These results suggest that LINC00426 could be also a biomarker for the functional immune status in PAM50 LB BRCA.

### 3.5 The validation of LINC00426 expression in an independent cohort supports its role as an immune phenotype-related biomarker and an OS prognostic marker in PAM50 LB BRCA

Next, we aimed to validate these results in a clinical independent cohort, the GEO-GSE96058 dataset (*n* = 3,052). The clinicopathological characteristics of PAM50 BRCA patients from this cohort are described in Supplementary Table S3. In this dataset, we identified that LINC00426 expression was significantly different between LA and HER2-enriched (*p* < 0.0001), LA and BL (*p* < 0.0001), LB and HER2-enriched (*p* < 0.0001), and LB and BL BRCA subtypes (*p* < 0.0001) (Supplementary Figure S1A). Kaplan-Meier analyses validated that LINC00426 expression did not have a prognostic value associated with OS in LA BRCA patients (*p* = 0.98) (Supplementary Figure S1B), while the high and low expression of LINC00426 were associated with increased and reduced OS in LB BRCA patients, respectively (*p* = 0.042) (Supplementary Figure S1C). In contrast to the BRCA-TCGA cohort, the LINC00426 expression was a prognostic marker for OS in HER2-enriched (*p* = 0.005) and BL (*p* = 0.005) BRCA patients in the GEO-GSE96058 cohort (Supplementary Figures S1D, E). These findings were validated via univariate Cox regression analyses (Supplementary Table S4). Multivariate Cox regression analyses showed that the OS prognostic value of LINC00426 expression in HER2-enriched and BL subtypes remained significant, while a tendency in LB subtype was observed in the GEO-GSE96058 cohort (Supplementary Tables S5–S7). In addition, we found that age (≤58 years old) and positive lymph node status were good and poor prognostic factors associated with OS, respectively, in all BRCA subtypes, which supports our findings in the BRCA-TCGA cohort (Supplementary Table S4).

As the LINC00426 expression was consistently identified as an OS prognostic marker in PAM50 LB subtype, we aimed to confirm whether LB BRCA patients with high and low expression of LINC00426 were associated with increased or reduced levels of tumor-infiltrating immune cell populations. In this context, we identified that LINC00426 expression differentially correlates with the infiltration level of 20 immune cell populations (Supplementary Table S8). Similar to our results in the BRCA-TCGA cohort, LB BRCA patients with low expression of LINC00426 showed low infiltration of naive B cells (*p* = 0.0003), plasma cells (*p* = 0.0225), CD8 T cells (*p* < 0.0001), memory CD4 T cells (resting) (*p* < 0.0001), memory CD4 T cells (activated) (*p* < 0.0001), gamma-delta T cells (*p* = 0.0004), M1 macrophages (*p* < 0.0001) and increased infiltration levels of memory B cells (*p* = 0.0049), NK cells (resting) (*p* < 0.0001), M0 macrophages (*p* = 0.0059), M2 macrophages (*p* < 0.0001), mast cells (resting) (*p* < 0.0001), mast cells (activated) (*p* < 0.0001) and eosinophils (*p* < 0.0001) (Supplementary Figure S2A). In addition, we found that LB BRCA patients with low expression of LINC00426 have reduced infiltration of T follicular helper cells (*p* = 0.0020), regulatory T cells (*p* < 0.0001), NK cells (activated) (*p* < 0.0001) and increased infiltration of naive CD4 T cells (*p* = 0.0137), dendritic cells (activated) (*p* = 0.0038) and neutrophils (*p* < 0.0001) (Supplementary Figure S2A), when compared to our results of the BRCA-TCGA cohort. We obtained the opposite results for the infiltration level of immune cell populations in the group of patients with high expression of LINC00426 (Supplementary Figure S2A). The infiltration of monocytes and dendritic cells (resting) did not show significant differences between groups of patients with low and high expression of LINC00426 in PAM50 LB BRCA (*p* > 0.05) (Supplementary Figure S2B).

We corroborated that LINC00426 expression positively correlates with the expression of *PDCD1* (R = 0.763), *PDCD1LG2* (R = 0.687), *CD274* (R = 0.648), *CTLA4* (R = 0.770), *LAG3* (R = 0.664), *TIGIT* (R = 0.807), *IDO1* (R = 0.737), *GZMA* (R = 0.783), *GZMB* (R = 0.705), *PRF1* (R = 0.763), ICG (R = 0.792) and CARG signatures (R = 0.773) (*p* < 0.001) (Supplementary Figure S3). The expression of ICG and CARG signatures were found to be significantly decreased in LB BRCA patients with low expression of LINC00426, in contrast to the high expression group (*p* < 0.0001) (Supplementary Figures S4A, B). Also, the expression of ICG and CARG signatures were positively correlated in PAM50 LB BRCA of the GEO-GSE96058 cohort (*p* < 0.001) (Supplementary Figure S4C). These results support our findings in the BRCA-TCGA cohort and highlight the consistent OS prognostic value of the LINC00426 expression and its relationship with the PAM50 LB BRCA immunobiology, suggesting a fundamental role in this subtype.

## 4 Discussion

The expression of lncRNAs vary between different cancer types and can promote or antagonize tumor progression (Qiu et al., 2013; Bhan et al., 2017; Bolha et al., 2017); therefore, the lncRNAs can be used as biomarkers for prognosis, treatment monitoring and as therapeutic molecular targets in cancer (Bolha et al., 2017). Specifically, lncRNAs are relevant in cancer immunobiology and have been proposed as immune-related biomarkers in different cancer types (Denaro et al., 2019; Zhang L. et al., 2020; Wu et al., 2020), including BRCA (Pei et al., 2018; Zhang Y. et al., 2020; DeVaux et al., 2020; Liu et al., 2020; Zhou et al., 2020; Zhang et al., 2021). Despite these advances, research on immune-related lncRNAs in PAM50 BRCA subtypes is limited. LINC00426 is an intergenic lncRNA located on 13q12.3 region (GeneCards, 2021) and has been studied in ccRCC, HCC, LUAD, NSCLC and OSA (Wang L. et al., 2020; Du, 2020; Zhu et al., 2020; Xiang et al., 2021). To our knowledge, this is the first study which evaluates the prognostic and biological role of LINC00426 in PAM50 BRCA subtypes.

In this study, we found that LINC00426 expression is a consistent OS predictor in PAM50 LB BRCA in the BRCA-TCGA and GEO-GSE96058 cohorts, in contrast to other subtypes. Particularly, the low and high expression of LINC00426 was associated with reduced and increased OS in LB BRCA patients, respectively. Interestingly, a previous study showed a similar prognostic behavior for LINC00426 in LUAD and NSCLC (Du, 2020). In contrast, Wang et al. reported that the high and low expression of LINC00426 is associated with reduced and increased OS in OSA, respectively (Wang Y. et al., 2020). We propose that LINC00426 expression could have a cancer type-dependent prognostic role. This hypothesis is supported by our pan-cancer exploratory analysis, where LINC00426 shows prognostic variations for OS in head and neck squamous cell carcinoma and hepatocellular carcinoma (Supplementary Figure S5). Similarly, a previous study showed dual prognostic roles of LINC00460 in different cancer types (Cisneros-Villanueva et al., 2021). Future studies might consider evaluating the prognostic role of LINC00426 between diverse cancer subtypes to determine potential differences, as we identified between PAM50 BRCA subtypes.

Previous reports demonstrated that LINC00426 promotes LUAD progression and doxorubicin resistance in OSA, suggesting a potential oncogenic role of LINC00426 in these cancers (Wang L. et al., 2020; Du, 2020). Conversely, Xiang et al. reported that LINC00426 expression positively correlates with CD8 T cells, while negatively correlates with monocytes and mast cells (resting) fractions in ccRCC (Xiang et al., 2021). We identified a differential correlation and infiltration changes between the LINC00426 expression and diverse immune cell populations in PAM50 LB BRCA patients, where the results for 14 immune cell populations were shared between BRCA-TCGA and GEO-GSE96058 cohorts. We propose that low expression of LINC00426 is potentially related with immunosuppressive TIMEs with high fractions of immune cell populations associated with cancer progression and immune evasion, such as mast cells, M0 and M2 macrophages (Stanton and Disis, 2016; Bense et al., 2017) in PAM50 LB BRCA, which potentially could be related with deficiencies in the host’s anti-tumor immune response. In contrast, we suggest that high expression of LINC00426 is potentially related with inflammatory TIMEs enriched with anti-tumoral immune cells, such as memory CD4 T cells, CD8 T cells and M1 macrophages (Stanton and Disis, 2016; Bense et al., 2017). This hypothesis is supported by our functional annotation analyses, where diverse immune-related processes were enriched in PAM50 LB BRCAs with high expression of LINC00426, indicating a favorable host’s anti-tumor immune response. Zhang et al. identified that the lncRNA TCL6 is correlated with the infiltration of B cells, CD8 T cells, CD4 T cells, neutrophils and dendritic cells, showing a prognostic value restricted for LB BRCA (Zhang Y. et al., 2020). Additional studies reported relationships between lncRNAs and immune cell infiltration in cancer (Li et al., 2020b; Liu et al., 2020; Zhang et al., 2021). Our findings are supported by previous studies that identified that the cell composition and functionality of tumor-immune cell infiltrates are strongly associated with diverse clinical outcomes in patients across different BRCA subtypes (Stanton and Disis, 2016; Bense et al., 2017; Vingiani et al., 2020).

Several studies demonstrated that tumor-intrinsic factors, like dysregulations on diverse oncogenic pathways, modulate the host’s anti-tumor immune response depending on the cancer type and cellular context (Cullis et al., 2018; Spranger and Gajewski, 2018; Pereira et al., 2022). We identified that PAM50 LB BRCAs with high expression of LINC00426 are also enriched with different cancer-related processes, such as KRAS signaling up, epithelial-mesenchymal transition and PI3K-AKT-mTOR. Tokumaru et al. demonstrated that the enrichment of KRAS signaling is associated with improved survival and favorable TIMEs enriched with B cells, CD8 T cells, M1 macrophages and monocytes in triple negative breast cancer (Tokumaru et al., 2020). Similarly, previous research identified that altered patterns of epithelial–mesenchymal transition markers are associated with inflammatory cell infiltrates in BRCA subtypes (Khadri et al., 2021). A study by Mafia et al. demonstrated that PI3K-AKT-mTOR signaling pathway is involved in the regulation of trafficking and functional roles of immune cells in the TIME (Mafi et al., 2022). Conversely, other studies found that apoptosis is associated with immune cell infiltration and cytolytic activity in BRCA (Rooney et al., 2015; Murthy et al., 2021), which supports our findings in the group of patients with high expression of LINC00426, where apoptosis is enriched and the CARG expression is increased. The differences in the enrichment of specific immune-related and cancer-related processes could explain the immune phenotypes and OS differences between PAM50 LB BRCA patients with low and high expression of LINC00426.

Because LINC00426 is related with immune cell infiltration, as detected in our functional annotation and CIBERSORTx analyses, we suggest that this lncRNA could be related with pathways involved in the recruitment of immune cell populations to the TIME (i.e., cytokines, chemokines and cell-cell adhesion pathways). In addition, previous studies showed relationships between lncRNAs, including FENDRR and BCAR4, with the expression of cytokines and chemokines in cancer, which could modulate the infiltration of immune cells to the TIME (Xing et al., 2014; Munteanu et al., 2021). Chen et al. demonstrated that the lncRNA LNMAT1 activates the expression of *CCL2* through epigenetic pathways and hnRNPL binding to the promotor region of *CCL2*, which results in the recruitment of tumor-associated macrophages to the TIME of bladder cancer, promoting lymphatic metastasis via VEGF-C excretion (Chen C. et al., 2018). Further functional studies might address the exact mechanisms of LINC00426 in the immune cell population’s recruitment in PAM50 LB BRCA.

Diverse studies revealed that the expression of immune-checkpoints (i.e., CTLA-4, PD-1 and PD-L1) and cytolytic activity markers (i.e., *GZMA* and *PFR1*) are important to determine the functional status of local anti-tumor immune response (Rooney et al., 2015; Sharma and Allison, 2015; Charoentong et al., 2017; Cogdill et al., 2017; Nishino et al., 2017; Narayanan et al., 2018; Thorsson et al., 2018; He and Xu, 2020). Reports suggest that lncRNAs could be related with the expression of these markers in cancer (Kathuria et al., 2018; Wang et al., 2019; Wei et al., 2019; Peng et al., 2020; Salama et al., 2020; Samir et al., 2021) with Xiang et al. having shown that LINC00426 correlates with *PDCD1* expression in ccRCC (Xiang et al., 2021). We identified that LINC00426 expression positively correlates with the expression of different ICGs (*PDCD1, PDCD1LG2, CD274, CTLA4, LAG3, TIGIT, IDO1*) and CARGs (*GZMA, GZMB, PRF1*). We suggest that LINC00426 could be involved, directly or indirectly, in the regulation of ICGs and CARGs expression in PAM50 LB BRCA. Studies demonstrated that the lncRNAs XIST, TSIX and MALAT1 regulate the PD-L1 expression in BRCA through ceRNA networks (Salama et al., 2020; Samir et al., 2021). Additional functional studies might elucidate the exact mechanisms of LINC00426 in the regulation of ICGs and CARGs expression in PAM50 LB BRCA.

Although the importance of the immune response was reported in LB BRCA (Nelson et al., 2017), there are currently no approved immunotherapies for the treatment of this subtype; however, some studies suggested that LB BRCA could be a potential candidate for immunotherapies (Bense et al., 2017; Nelson et al., 2017; Griguolo et al., 2021). Zhu et al. proposed that luminal BRCAs could be stratified in three different immune subtypes based on the expression of immune-related genes (Zhu et al., 2019). Food and Drug Administration (FDA) clinical trials like NCT04659551, NCT03356860 and NCT03815890 are currently evaluating the use of immune checkpoint inhibitors (i.e., Durvalumab, Nivolumab and Ipilimumab) in LB BRCA. In this context, the use of immune-related lncRNAs, such as LINC00426, might be useful for identifying patients who could benefit from immunotherapies, expanding the treatment options for LB BRCA.

The main limitations of our study are related to its retrospective nature and bioinformatics approach based on transcriptomic data, limiting the mechanistic conclusions of LINC00426. Validation of these findings is needed through methodologies like multiplex immunofluorescence or flow cytometry. Future studies focused on LINC00426 in PAM50 LB BRCA are needed that include experimental approaches to gain a wide understanding about the exact functional role of LINC00426. Despite these limitations, we conclude that LINC00426 is a potential biomarker of cancer immune phenotype whose expression has a consistent and an OS prognostic value in PAM50 LB BRCA patients in two independent cohorts, which suggest a potential use for immunotherapies selection in patients, but further analyses are mandatory to confirm this hypothesis.

## Data Availability

The datasets presented in this study can be found in online repositories. The names of the repositories and accession numbers can be found in the article and Supplementary Material.
