# Supplementary material for: LINC00426 is a potential immune phenotype-related biomarker and an overall survival predictor in PAM50 luminal B breast cancer
Source: Front Genet. 2023 May 16;14:1034569. doi: 10.3389/fgene.2023.1034569 (PMC10228735; doi:10.3389/fgene.2023.1034569)
Supplement: Supplementary file 1 [file DataSheet1.PDF]

Supplementary Material

Supplementary Table 1. Top 5 of differentially expressed genes (up- and down-regulated) between PAM50 LB BRCA patients with low and high expression of LINC00426.

Gene symbol	Gene name	Pathway	log_2FC (high vs low expression of LINC00426)	Adj. p value
CPB1	Carboxypeptidase B1	Proteolysis	7.470	9.910×10^{-25}
TRH	Thyrotropin Releasing Hormone	Histamine metabolic process	7.020	1.020×10^{-37}
SYT4	Synaptotagmin 4	Exocytosis	7.010	1.070×10^{-7}
CPLX2	Complexin 2	Exocytosis	6.360	3.830×10^{-18}
NELL1	Neural EGFL Like 1	Regulation of gene expression	5.280	1.570×10^{-9}
CLEC6A	C-Type Lectin Domain Containing 6A	Positive regulation of cytokine production	-5.090	2.270×10^{-30}
IFNG	Interferon Gamma	Positive regulation of cytokine production	-5.340	1.580×10^{-46}
PLA2G2D	Phospholipase A2 Group IID	CD4-positive, CD25-positive, alpha-beta regulatory T cell differentiation	-5.830	2.370×10^{-59}
DCD	Dermcidin	Proteolysis	-6.430	1.300×10^{-10}
GNAT3	G Protein Subunit Alpha Transducin 3	G protein-coupled receptor signaling pathway	-6.580	1.960×10^{-5}

FC: fold change.

Supplementary Table 2. Spearman correlation of LINC00426 expression with the infiltration level of 22 immune cell populations in PAM50 LB patients from the BRCA-TCGA cohort.

Immune cell population	p value	Spearman correlation value
CD8 T cells	2.230×10^{-20}	0.603
Memory CD4 T cells (activated)	6.740×10^{-15}	0.523
M2 macrophages	7.210×10^{-12}	-0.468
NK cells (resting)	2.370×10^{-9}	-0.414
M0 macrophages	2.630×10^{-9}	-0.413
Gamma-delta T cells	6.500×10^{-9}	0.403
M1 macrophages	1.140×10^{-8}	0.397
Memory B cells	7.230×10^{-8}	-0.377
Eosinophils	1.160×10^{-5}	-0.311
Mast cells (activated)	4.740×10^{-5}	-0.289
Naive B cells	0.002	0.222
Memory CD4 T cells (resting)	0.003	0.201
Mast cells (resting)	0.006	-0.198
Plasma cells	0.010	0.186
T follicular helper cells	0.043	0.146
Naive CD4 T cells	0.086	-0.124
Monocytes	0.167	-0.100
Neutrophils	0.347	-0.068
Dendritic cells (activated)	0.755	0.023
Regulatory T cells	0.797	0.019
NK cells (activated)	0.893	0.010
Dendritic cells (resting)	0.930	0.006

Supplementary Table 3. Clinicopathological characteristics of luminal A, luminal B, HER2-enriched and basal-like BRCA patients from the GEO-GSE96058 cohort (n = 3052).

		**Luminal A (n = 1657)**	**Luminal B (n = 729)**	**HER2-enriched (n = 327)**	**Basal-like (n = 339)**
**Variable**	**Stratification**	**Frequency (n)**	**Frequency (n)**	**Frequency (n)**	**Frequency (n)**
Age	≤58	580	518	127	170
	>58	1077	211	200	169
Lymph node status	Positive	541	292	155	98
	Negative	1071	421	153	231
	NA	45	16	19	10
ER status	Positive	1633	727	198	80
	Negative	9	1	69	148
	NA	15	1	60	111
PR status	Positive	1542	641	140	68
	Negative	49	42	106	155
	NA	66	46	81	116
HER2 status	Positive	83	84	205	20
	Negative	1505	625	112	305
	NA	69	20	10	14
OS status	Alive	1541	647	272	272
	Dead	116	82	55	67

BRCA: breast cancer; ER: estrogen receptor; HER2: human epidermal growth factor receptor 2; NA: not available; OS: overall survival; PR: progesterone receptor.

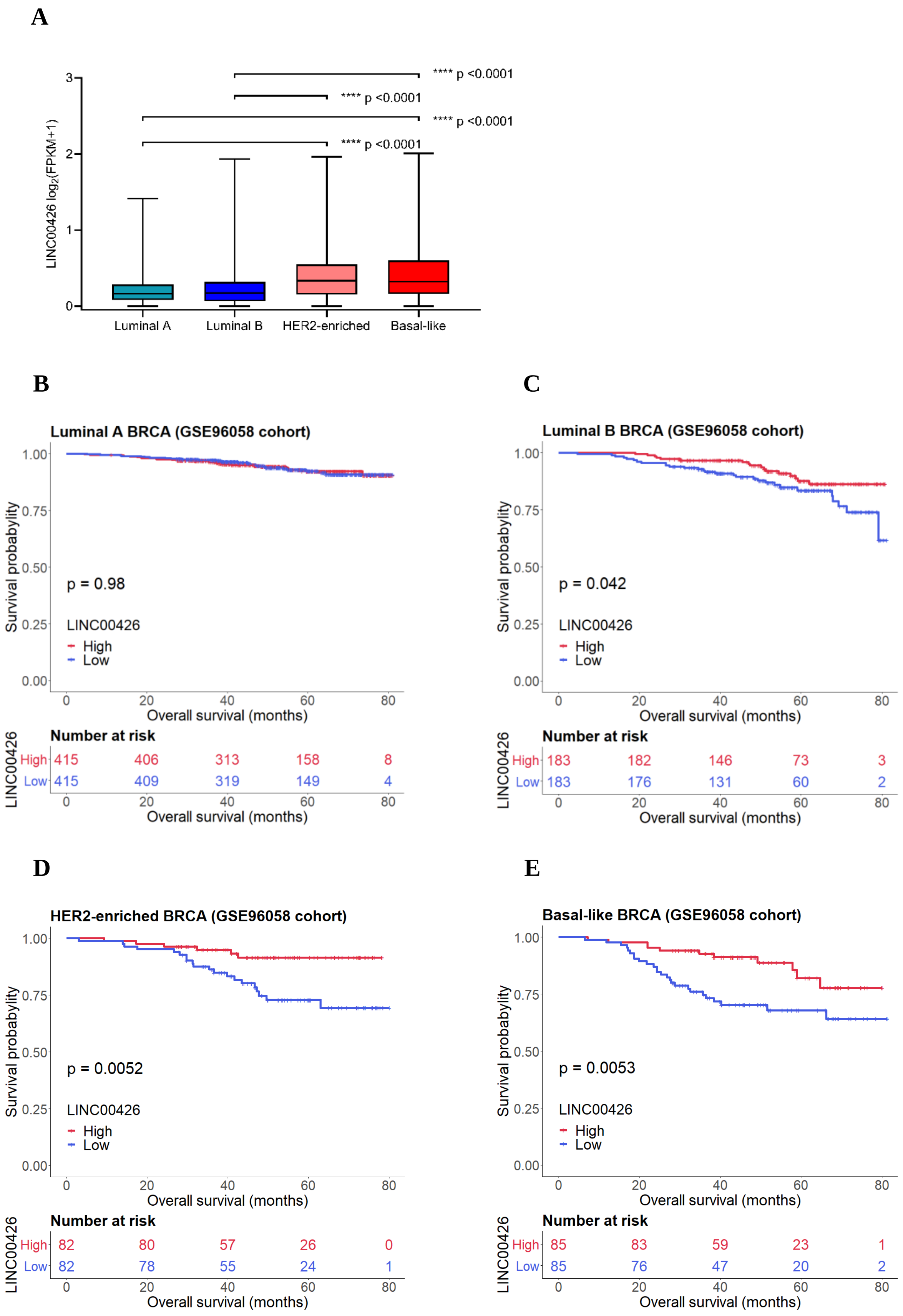
A
LINC00426 log2(FPKM+1)
**** p <0.0001
**** p <0.0001
**** p <0.0001
**** p <0.0001
Luminal A
Luminal B
HER2-enriched
Basal-like
B
Luminal A BRCA (GSE96058 cohort)
Survival probability
p = 0.98
LINC00426
High
Low
Overall survival (months)
Number at risk
High 415 406 313 158 8
Low 415 409 319 149 4
C
Luminal B BRCA (GSE96058 cohort)
p = 0.042
Number at risk
High 183 182 146 73 3
Low 183 176 131 60 2
D
HER2-enriched BRCA (GSE96058 cohort)
p = 0.0052
Number at risk
High 82 80 57 26 0
Low 82 78 55 24 1
E
Basal-like BRCA (GSE96058 cohort)
p = 0.0053
Number at risk
High 85 83 59 23 1
Low 85 76 47 20 2

Supplementary Figure 1. LINC00426 expression and Kaplan-Meier survival analyses in PAM50 BRCA patients from the GEO-GSE96058 cohort. (**A**) Expression level of LINC00426 in BRCA patients stratified by PAM50 subtypes (Kruskal-Wallis test) (luminal A: n = 1657; luminal B: n = 729; HER2-enriched: n = 327; and basal-like: n = 339). (**B**) Kaplan-Meier survival plot of the LINC00426 expression in the OS of luminal A (n = 1657), (**C**) luminal B (n = 729), (**D**) HER2-enriched (n = 327) and (**E**) basal-like (n = 339) BRCA patients (p <0.05).

Supplementary Table 4. Univariate Cox proportional hazard regression analyses of clinicopathological variables impacting in the OS of luminal A, luminal B, HER2-enriched and basal-like BRCA patients (GEO-GSE96058 cohort), including the LINC00426 expression. Bold indicates p values <0.05. A variable with HR <1 indicates a poor prognostic factor, while a variable with HR >1 indicates a good prognostic factor.

**Variables**	**Luminal A (n = 1657)**		**Luminal B (n = 729)**	
	**HR (95% CI)**	**p value**	**HR (95% CI)**	**p value**
Age (≤58)	**0.151 (0.076-0.297)**	**< 0.001**	**0.243 (0.117-0.504)**	**< 0.001**
Lymph node status (positive)	1.106 (0.749-1.633)	0.613	1.152 (0.731-1.814)	0.543
ER status (positive)	1.212×10^6 (0.0-Inf)	0.993	1.207×10^6 (0.0-Inf)	0.996
PR status (positive)	0.610 (0.249-1.496)	0.280	0.541 (0.248-1.180)	0.122
HER2 status (positive)	1.184 (0.551-2.542)	0.666	0.757 (0.365-1.570)	0.454
LINC00426 expression (low)	0.995 (0.577-1.713)	0.985	**1.843 (1.013-3.356)**	**0.045**

**Variables**	**HER2-enriched (n = 327)**		**Basal-like (n = 339)**	
	**HR (95% CI)**	**p value**	**HR (95% CI)**	**p value**
Age (≤58)	**0.327 (0.165-0.648)**	**0.001**	**0.465 (0.279-0.774)**	**0.003**
Lymph node status (positive)	**2.983 (1.586-5.612)**	**< 0.001**	**2.008 (1.234-3.265)**	**0.005**
ER status (positive)	0.886 (0.430-1.825)	0.743	1.405 (0.740-2.667)	0.299
PR status (positive)	0.896 (0.449-1.791)	0.757	0.812 (0.393-1.677)	0.573
HER2 status (positive)	0.765 (0.447-1.308)	0.327	0.476 (0.116-1.943)	0.301
LINC00426 expression (low)	**3.401 (1.366-8.470)**	**0.008**	**2.628 (1.298-5.322)**	**0.007**

BRCA: breast cancer; CI: confidence interval; HR: hazard ratio.

Supplementary Table 5. Multivariate Cox proportional hazard regression analysis of clinicopathological variables impacting in the OS of luminal B BRCA patients (GEO-GSE96058 cohort). Bold indicates p values <0.05. A variable with HR <1 indicates a poor prognostic factor, while a variable with HR >1 indicates a good prognostic factor.

Variables	Luminal B (n = 729)	
	HR (95% CI)	p value
**Age (≤58)**	**0.281 (0.111-0.714)**	**0.008**
LINC00426 expression (low)	1.702 (0.933-3.102)	0.082

BRCA: breast cancer; CI: confidence interval; HR: hazard ratio.

Supplementary Table 6. Multivariate Cox proportional hazard regression analysis of clinicopathological variables impacting in the OS of HER2-enriched BRCA patients (GEO-GSE96058 cohort). Bold indicates p values <0.05. A variable with HR <1 indicates a poor prognostic factor, while a variable with HR >1 indicates a good prognostic factor.

Variables	HER2-enriched (n = 327)	
	HR (95% CI)	p value
**Age (≤58)**	**0.328 (0.121-0.889)**	**0.028**
**Lymph node status (positive)**	**2.971 (1.216-7.260)**	**0.017**
**LINC00426 expression (low)**	**4.812 (1.772-13.068)**	**0.002**

BRCA: breast cancer; CI: confidence interval; HR: hazard ratio.

Supplementary Table 7. Multivariate Cox proportional hazard regression analysis of clinicopathological variables impacting in the OS of basal-like BRCA patients (GEO-GSE96058 cohort). Bold indicates p values <0.05. A variable with HR <1 indicates a poor prognostic factor, while a variable with HR >1 indicates a good prognostic factor.

Variables	Basal-like (n = 339)	
	HR (95% CI)	p value
**Age (≤58)**	**0.203 (0.087-0.473)**	**< 0.001**
**Lymph node status (positive)**	**2.869 (1.455-5.657)**	**0.002**
**LINC00426 expression (low)**	**2.344 (1.127-4.876)**	**0.023**

BRCA: breast cancer; CI: confidence interval; HR: hazard ratio.

Supplementary Table 8. Spearman correlation of LINC00426 expression with the infiltration level of 22 immune cell populations in PAM50 LB BRCA patients from the GEO-GSE96058 cohort.

Immune cell population	p value	Spearman correlation value
CD8 T cells	1.790×10^{-85}	0.641
M2 macrophages	9.320×10^{-61}	-0.557
M1 macrophages	1.060×10^{-58}	0.549
Memory CD4 T cells (resting)	2.830×10^{-34}	0.431
Neutrophils	2.470×10^{-29}	-0.400
NK cells (resting)	3.440×10^{-21}	-0.340
Regulatory T cells	1.720×10^{-20}	0.334
Memory CD4 T cells (activated)	2.550×10^{-14}	0.277
NK cells (activated)	3.230×10^{-14}	0.276
Mast cells (activated)	3.180×10^{-12}	-0.254
Eosinophils	3.230×10^{-11}	-0.242
Mast cells (resting)	1.610×10^{-10}	-0.234
Gamma-delta T cells	1.940×10^{-6}	0.175
Naive B cells	0.001	0.126
T follicular helper cells	0.001	0.119
Naive CD4 T cells	0.001	-0.117
Dendritic cells (activated)	0.002	-0.114
Plasma cells	0.002	0.112
Memory B cells	0.003	-0.111
M0 macrophages	0.007	-0.100
Monocytes	0.240	-0.044
Dendritic cells (resting)	0.850	-0.007

A

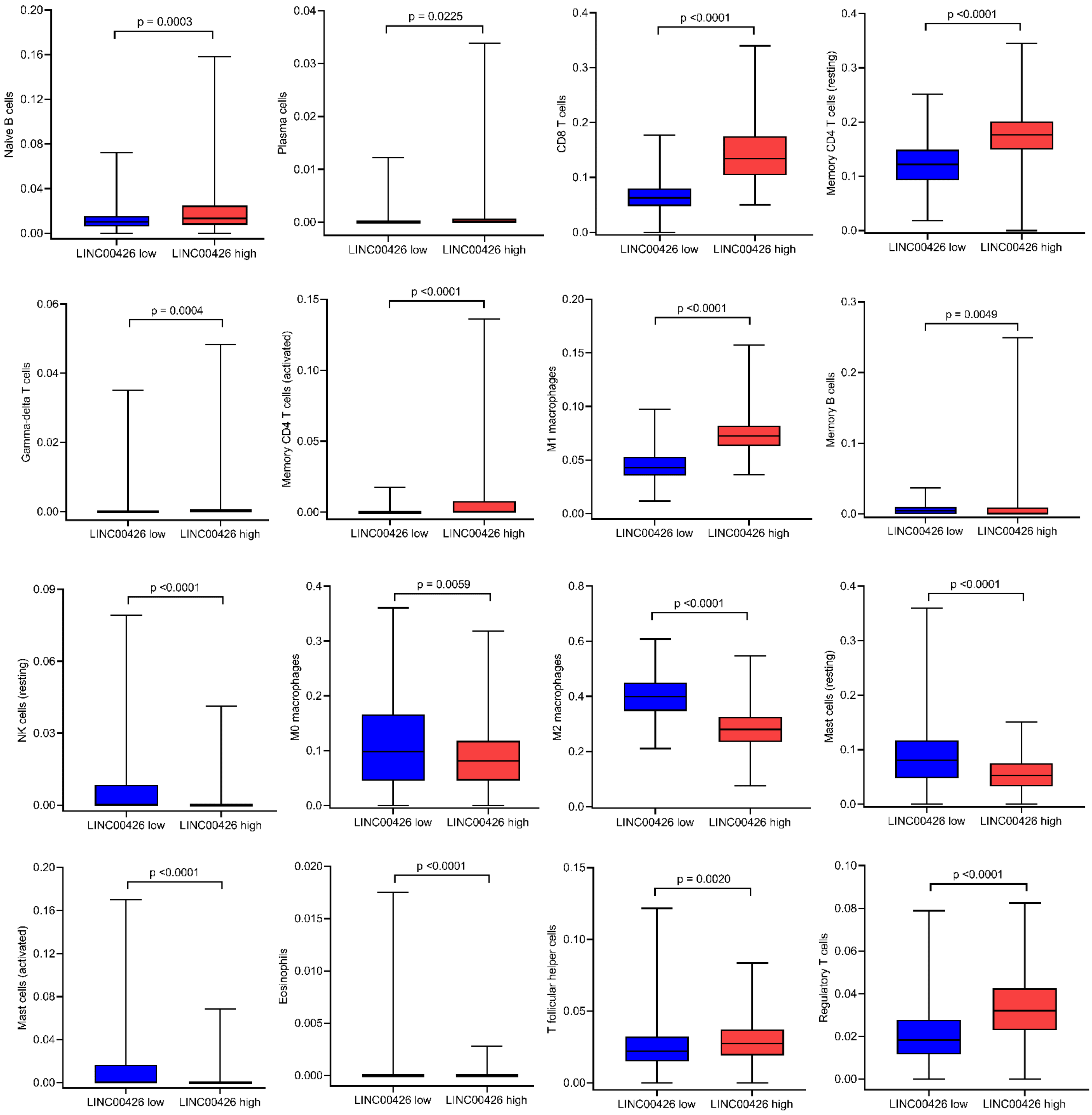
Naive B cells
p = 0.0003
Plasma cells
p = 0.0225
CD8 T cells
p <0.0001
Memory CD4 T cells (resting)
p <0.0001
Gamma-delta T cells
p = 0.0004
Memory CD4 T cells (activated)
p <0.0001
M1 macrophages
p <0.0001
Memory B cells
p = 0.0049
NK cells (resting)
p <0.0001
M0 macrophages
p = 0.0059
M2 macrophages
p <0.0001
Mast cells (resting)
p <0.0001
Mast cells (activated)
p <0.0001
Eosinophils
p <0.0001
T follicular helper cells
p = 0.0020
Regulatory T cells
p <0.0001
LINC00426 low
LINC00426 high

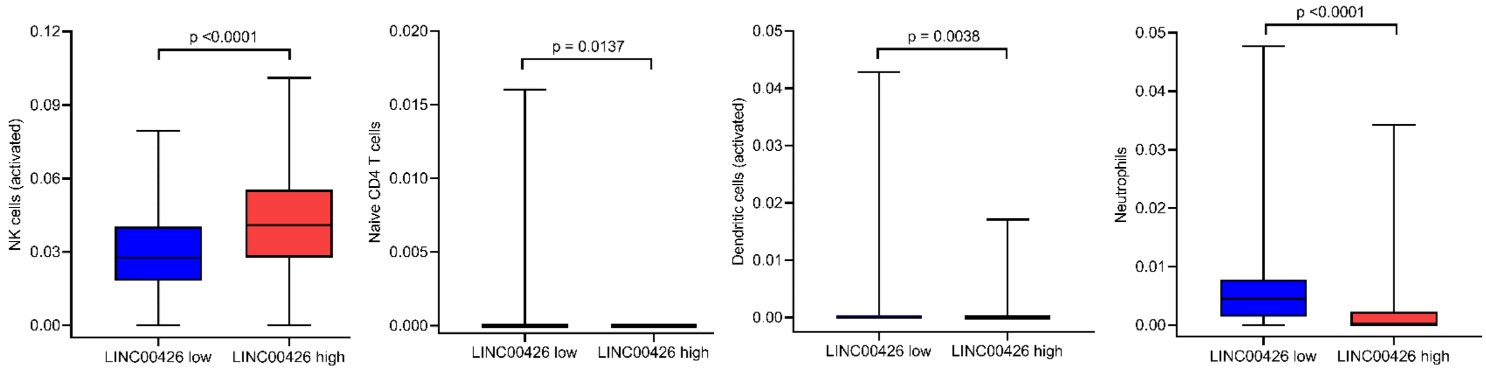

B

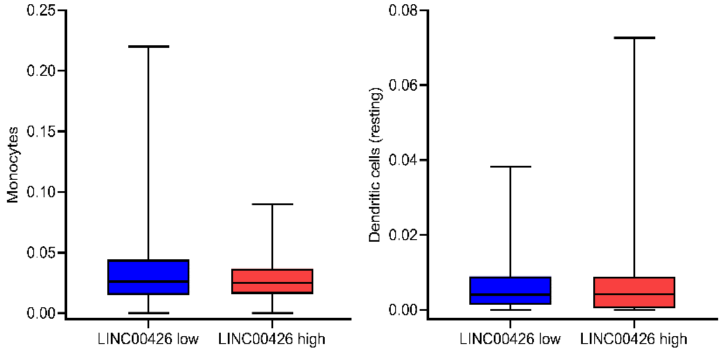

Supplementary Figure 2. Infiltration levels of immune cell populations in PAM50 LB BRCA patients with low and high expression of LINC00426 from the GEO-GSE96058 cohort. (**A**) Mann-Whitney U test shows significant differences (p <0.05) in the infiltration level of naive B cells, plasma B cells, CD8 T cells, memory CD4 T cells (activated), M1 macrophages, memory B cells, NK cells (resting), M0 macrophages, mast cells (resting), mast cells (activated), eosinophils, T follicular helper cells, regulatory T cells, NK cells (activated), naive CD4 T cells, dendritic cells (activated) and neutrophils. (**B**) Infiltration of monocytes and dendritic cells (resting) did not show significant differences (Mann-Whitney U test, p >0.05).

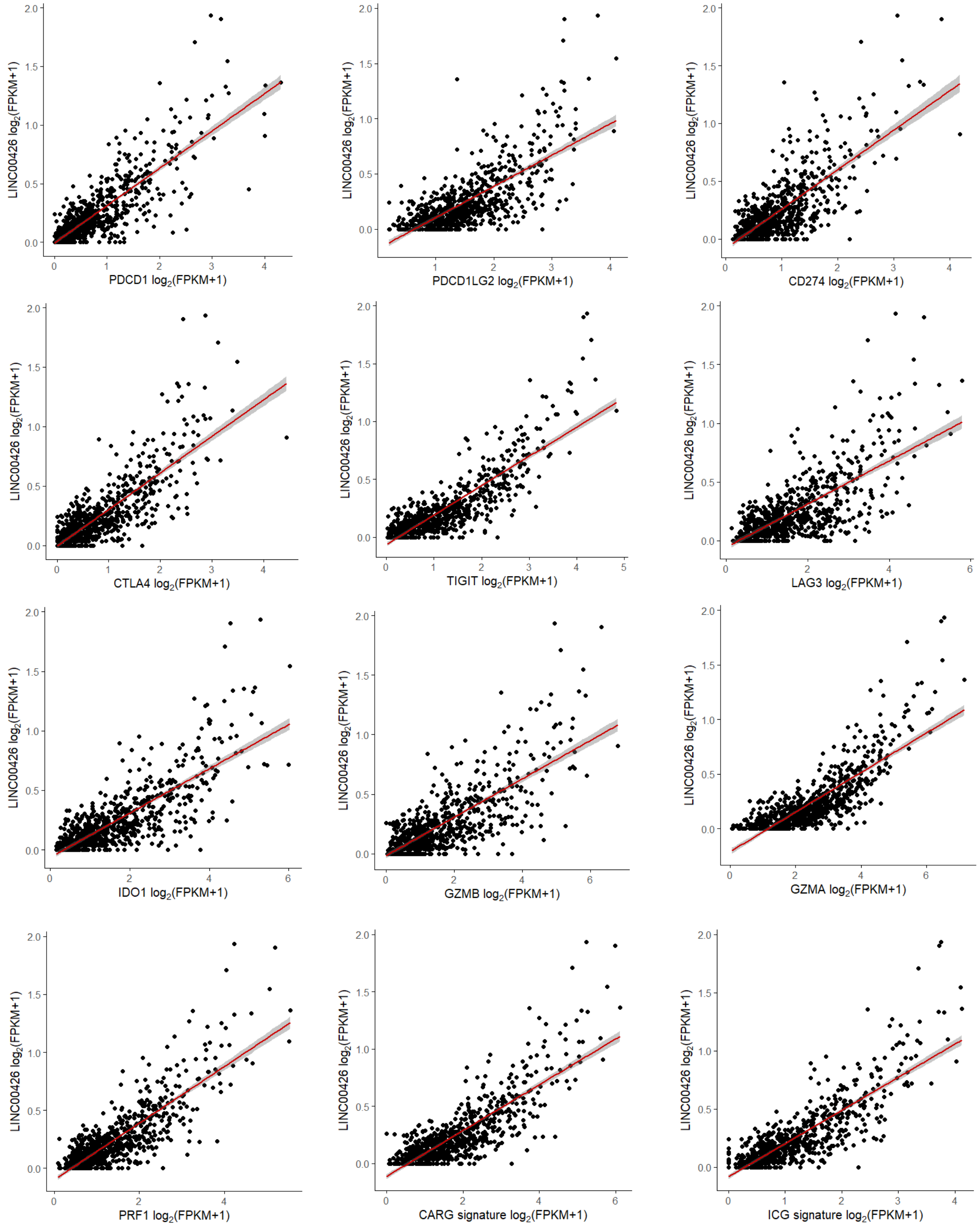

Supplementary Figure 3. Spearman correlation of the LINC00426 expression with ICG and CARG expression in PAM50 LB BRCA patients from the GEO-GSE96058 cohort. Positive correlation with statistical significance (p <0.001) was identified in *PDCD1*, *CD274*, *PDCD1LG2*, *CTLA4*, *LAG3*, *TIGIT*, *IDO1*, *GZMA*, *GZMB*, *PRF1*, *ICG* and CARG signatures.

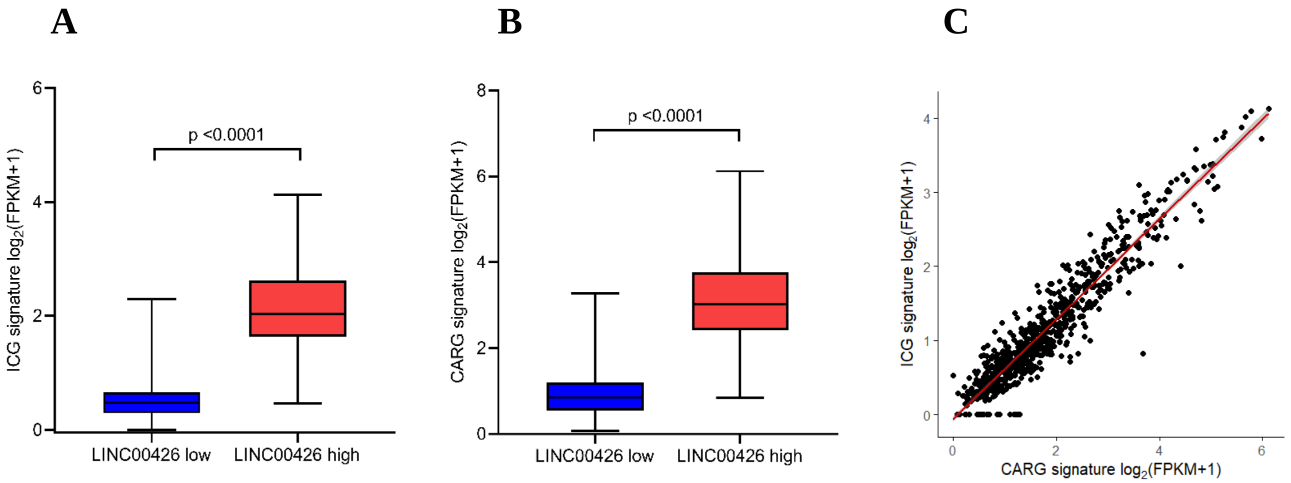

Supplementary Figure 4. ICG and CARG signature expression in PAM50 LB BRCA patients from the GEO-GSE96058 cohort. Differences in the (**A**) ICG and (**B**) CARG signature expression were detected between groups of patients with low and high expression of LINC00426 ($p < 0.05$). (**C**) Spearman correlation between ICG and CARG signature expression in PAM50 LB BRCA patients ($p < 0.001$).

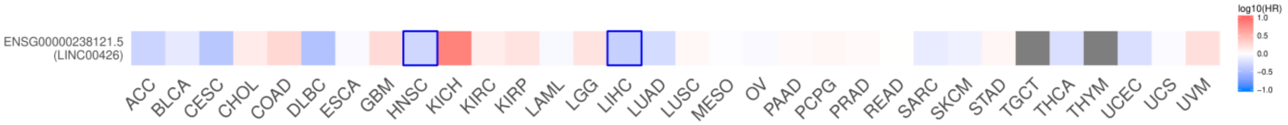

Supplementary Figure 5. OS analysis based on the LINC00426 expression across 32 cancer types from the TCGA in GEPIA2 platform. The heatmap depicts the Hazard Ratios ($\log_{10}$HR) of the expression of LINC00426 across 32 cancer types, where significant results were obtained in HNSC and LIHC (Mantel-Cox test, p adjusted value <0.05). The red and blue color represent poor and good prognosis, respectively. The color intensity indicates the HR values. Adrenocortical carcinoma (ACC); Bladder urothelial carcinoma (BLCA); Cervical squamous cell carcinoma and endocervical adenocarcinoma (CESC); Cholangiocarcinoma (CHOL); Colon adenocarcinoma (COAD); Diffuse large B-cell lymphoma (DLBCL); Esophageal carcinoma (ESCA); Glioblastoma multiforme (GBM); Head and neck squamous cell carcinoma (HNSC); Kidney chromophobe (KICH); Kidney renal clear cell carcinoma (KIRC); Kidney renal papillary cell carcinoma (KIRP); Acute myeloid leukemia (LAML); Low grade glioma (LGG); Liver hepatocellular carcinoma (LIHC); Lung adenocarcinoma (LUAD); Lung squamous cell carcinoma (LUSC); Mesothelioma (MESO); Ovarian serous cystadenocarcinoma (OV); Pancreatic adenocarcinoma (PAAD); Pheochromocytoma and paraganglioma (PCPG); Prostate adenocarcinoma (PRAD); Rectum adenocarcinoma (READ); Sarcoma (SARC); Skin cutaneous melanoma (SKCM); Stomach adenocarcinoma (STAD); Testicular germ cell tumors (TGCT); Thyroid carcinoma (THCA); Thymoma (THYM); Uterine corpus endometrial carcinoma (UCEC); Uterine carcinosarcoma (UCS); Uveal melanoma (UVM).
